# Development of a Portable Single Photon Ionization-Photoelectron Ionization Time-of-Flight Mass Spectrometer

**DOI:** 10.1155/2015/581696

**Published:** 2015-10-26

**Authors:** Yunguang Huang, Jinxu Li, Bin Tang, Liping Zhu, Keyong Hou, Haiyang Li

**Affiliations:** ^1^Electric Power Research Institute, Guangxi Power Grid Corporation, Nanning 530023, China; ^2^Key Laboratory of Separation Science for Analytical Chemistry, Dalian Institute of Chemical Physics, Chinese Academy of Sciences, Dalian 116023, China

## Abstract

A vacuum ultraviolet lamp based single photon ionization- (SPI-) photoelectron ionization (PEI) portable reflecting time-of-flight mass spectrometer (TOFMS) was designed for online monitoring gas samples. It has a dual mode ionization source: SPI for analyte with ionization energy (IE) below 10.6 eV and PEI for IE higher than 10.6 eV. Two kinds of sampling inlets, a capillary inlet and a membrane inlet, are utilized for high concentration and trace volatile organic compounds, respectively. A mass resolution of 1100 at *m/z* 64 has been obtained with a total size of 40 × 31 × 29 cm, the weight is 27 kg, and the power consumption is only 70 W. A mixture of benzene, toluene, and xylene (BTX), SO_2_, and discharging products of SF_6_ were used to test its performance, and the result showed that the limit of quantitation for BTX is as low as 5 ppbv (*S*/*N* = 10 : 1) with linear dynamic ranges greater than four orders of magnitude. The portable TOFMS was also evaluated by analyzing volatile organic compounds from wine and decomposition products of SF_6_ inside of a gas-insulated switchgear.

## 1. Introduction

Mass spectrometry has been widely employed for detection of environmental pollutants, illegal drugs, and explosives for its rapid, high sensitivity, and accuracy in qualitative analysis [1]. However, traditional mass spectrometry used in lab can barely be applied for in situ detection due to its size and weight. The technology of portable mass spectrometry has been recognized as one of the most promising and widely used techniques in environment monitoring, field diagnosis, and process monitoring.

Researches on miniaturization of magnetic mass spectrometry, quadrupole mass spectrometry, ion trap mass spectrometry, and time-of-flight mass spectrometry (TOFMS) all have been reported in the literature [2–8]. Compared with other types of mass spectrometry, TOFMS provides rapid scanning speed, full range scanning of the mass to charge, and simpleness in structure. Particularly, TOFMS can give a satisfying performance with moderate mechanical accuracy. Hopkins et al. have achieved remarkable approaches in developing and application of field detecting TOFMS [9–11]. A tiny TOFMS analyzer was developed with a length of 20 cm and 500 g in weight. A coaxial reflecting TOFMS with MALDI ion source was also developed, and the mass range is as high as 10,000* m/z*; sensitivity of picomole and resolution of 300–1000 (*m*/Δ*m*) were achieved. Cornish and Cotter [12] designed an end cap miniature TOFMS, this TOFMS featured with a laser ion source and a resolution of 210 at 1061* m/z*. Brinckerhoff et al. [13] explored a laser-focusing micro TOFMS with coaxial structure and a length of 20 cm, and multifield energy focusing technique was applied to this micro TOFMS. The micro TOFMS has a resolution of 1000 *m*/Δ*m* with an upper mass range to 1000* m/z* and was used for elemental and organic analysis on the surface of a planet. White et al. developed a mini TOFMS with membrane inlet and EI ion source [14]. The mini TOFMS was 53 × 33 × 21 cm in size and 21 kg in weight with a resolution of 300 at 28* m/z*. The experiment data was well linear fitted in the range of 50–1200 ppb for BTX. A portable TOFMS investigated by Syage et al. has a frequency of 200 Hz [15], and the limit of detection for aromatic compounds was 10–100 ppb with 10 s analysis time.

In situ portable TOFMS prefers soft ionization source for rapid spectrum interpretation. Due to the 70 eV high energy electrons, a large number of ion fragments could be present with traditional electron ionization source, and the overlapping peaks from fragments ions would bring much trouble in spectrum interpretation. A VUV lamp is a type of soft ionization source, and the analyte molecules can be ionized through single photon ionization (SPI) with few fragments. The VUV lamp is compact and can be operated without chemical reagents. Therefore, the VUV lamp is very suitable for rapid in situ detection. Li et al. have been working on developing portable and miniature TOFMS with VUV lamp ever since 2002, such as SPI source with membrane inlet miniature TOFMS, magnetic enhanced photon ionization source TOFMS, single photon ionization combined with chemical ionization source TOFMS, and quasi-trapping chemical ionization source TOFMS [16–19]. Proton transfer reaction mass spectrometer (PTRMS) is another widely used soft ionization source for online measurements of trace components with concentrations as low as a few pptv, which is developed on the basis of proton transfer reactions with reactant ion of H_3_O^+^. But the PTR reaction is fulfilled at high pressure, and the vacuum system is much larger in the PTRMS, so it is very hard for the miniaturization with PTRMS [20, 21].

The unique characteristics of the present portable TOFMS compared to existing technologies are the combination of novel dual SPI/PEI ionization source, versatile sampling inlet, differential pumping for high gas throughput, and low-power consumption. The details of the operating conditions and the applications of the portable TOFMS were demonstrated in the following sections.

## 2. Description of Spectrometer

### 2.1. Instrument Description

**Figure 1:**
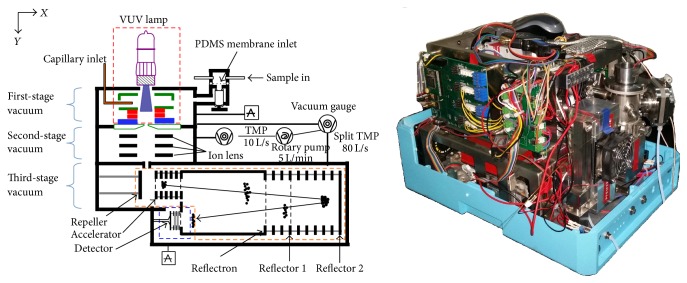
Schematic diagram and photograph of the portable time-of-flight mass spectrometer.

A schematic diagram and photograph that illustrate the features of the portable TOFMS are presented in Figure 1. The dimensions of the TOFMS are 40 × 31 × 29 cm, and it weighs about 27 kg including the battery and all the pumping system. Power consumption is approximately 70 W at working condition. The portable TOFMS consists of an ionization source, an orthogonal acceleration reflection mass analyzer, two sampling inlets, the pumping system, and the electronics for the TOFMS timing and data acquisition. The ions generated from ionization source are accelerated from the source region to orthogonal repelling region through a set of electrodes. The repeller then was applied with pulse voltage to converge the ions into acceleration region, and then the ions enter a field-free region. A cylindrical reflectron is used for focusing the ion beam and directing it back along the flight tube. When the ion beam reaches the detector, it is detected by a micro-channel-plate detector. The electronic signal from the detector is sent to a time-to-digital converter (TDC) based data acquisition system. Mass spectrum histogram with 100 ps resolution is then passed to a laptop PC. Data analysis and processing including data reduction are performed using home-made data processing software.

The portable TOFMS employs a three-stage differential pumping system with an 80 L/s split-flow turbomolecular pump and a 10 L/s turbomolecular pump; both of the turbomolecular pumps are backed by a 5 L/min diaphragm pump. The 10 L/s turbomolecular pump is used for the first-stage differential pumping of ion source and held the pressure at 1.5 Pa. The split-flow turbomolecular pump provides about 3 L/s pumping for second stage of ion transmission region and 80 L/s pumping for the third stage of mass analyzer (kept below 5 × 10^−4^ Pa at working condition). Vacuum gauges (MKS 925) were used to monitor the pressure of ion source, and a MKS 972 vacuum gauge was used for mass analyzer.

### 2.2. Sampling Inlet

The portable TOFMS is equipped with two kinds of sampling inlet: a fused silica capillary (150 *μ*m in i.d., 1.7 m in length) based direct sampling inlet for high concentration analytes and a sheet PDMS membrane (with thickness of 50 *μ*m and 150 mm^2^ in area) inlet for trace analytes. A stainless steel mesh was set under the membrane to keep the sheet membrane flat. Volatile organic compounds (VOCs) in the sample diffuse faster than air when passing through the membrane into the ionization region; therefore, the VOCs are relatively enriched. The flow rate of the samples through the surface of the membrane was optimized as 1 L/min and was controlled by a mass flow controller (Seven Star Electronics Co., Ltd., Beijing, China).

### 2.3. Ionization Source and Ion Transmission Region

**Figure 2:**
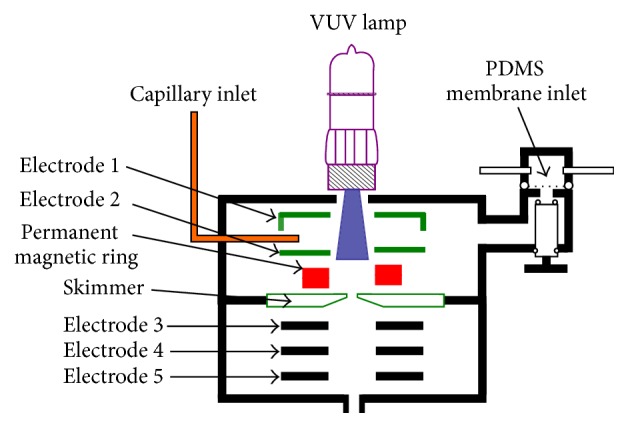
Configurations of the electrodes inside of the ion source and ion transmission region.

The electrodes configuration inside of the ion source and ion transmission region is shown in Figure 2. The ionization source consists of three parts: a VUV lamp, permanent magnetic ring, and DC voltage lens including electrode 1, electrode 2, and skimmer electrode. The VUV lamp is a commercial low-pressure krypton discharge lamp (10.6 eV, Cathodeon Ltd., Cambridge, UK), with a flux of about 1 × 10^11^ photons s^−1^, and is installed on the top of ionization chamber. The VUV lamp was lighted by −1400 V DC voltages. The ion lens in the ion transmission region includes three cylinders: electrode 3, electrode 4, and electrode 5. Electrode 1, electrode 2, and skimmer are all tubular steel stainless electrodes; the size is 5.5 mm in length, 16 mm i.d., 28 mm o.d., and with 4 mm central hole (1 mm central hole for skimmer electrode). These three electrodes are separated by insulator rings, and the distance from the VUV lamp light window to the surface of orifice electrode is only 20 mm. The permanent magnetic ring locates between electrode 2 and skimmer, the dimensions were 31 mm in inner diameter, 19 mm in outer diameter, and 6.5 mm in thickness, and it offered a maximum energy product (BHmax) of 1200 kJ/m^3^.

The ion transmission region consists of three circular electrodes with inner diameter of 10 mm and thickness of 2 mm, 1 mm, and 2 mm, respectively. The insulated spaces among the skimmer, electrode 3, electrode 4, and electrode 5 are all 2 mm.

The ion source was operated at two modes: single photoionization (SPI) mode for analytes with ionization energy (IE) lower than 10.6 eV and photoelectron ionization (PEI) mode for analytes with IE higher than 10.6 eV. The two different modes were controlled by the DC voltage applied on electrodes inside of ionization source. When 20 V, 17 V, and 5 V were applied to electrode 1, electrode 2, and skimmer separately, the ionization source operates under SPI mode, and voltages on electrode 3, electrode 4, and electrode 5 were −20 V, −120, and 40 V. The PEI mode was originated from the electrons generated on the surface of skimmer by the photoelectric effect when VUV light irradiated on electrodes. The PEI mode was activated when voltages on electrode 1, electrode 2, and skimmer were changed to 22.5 V, 21 V, and −44.5 V. The energy of the photoelectron was calculated as 67 eV by subtracting the voltage on the skimmer from the voltage on electrode 1, and the voltages on electrode 3, electrode 4, and electrode 5 were changed to 9 V, −120, and 0 V, accordingly.

The total power consumption of the present ionization source was only 1.5 W with the VUV lamp, much lower than 50 W with traditional EI ionization source.

The mass analyzer of TOFMS consists of four parts: the ion repeller region, ion accelerator region, field-free region, and the reflectron with a length of 6 mm, 23 mm, 210 mm, and 58 mm, respectively. Typical parameters of the portable time-of-flight mass spectrometer were shown in Table 1.

### 2.4. Methods Used for Gas Preparation

The two standard gas mixtures of 1 ppmV benzene, toluene, and p-xylene (BTX) and 50 ppm SO_2_ diluted with N_2_ (99.9993% purity) were purchased from Dalian Special Gas Company (Dalian, China). The samples of BTX and SO_2_ with different concentrations used in the experiments were obtained by diluting standard gas with pure N_2_ within a PTFE sampling bag. The dilution process was as follows: a measured amount of N_2_ was blown into a PTFE sampling bag, and then a desired amount of sample was added to the bag. The amount of N_2_ and sample was controlled by 3 L/min and 100 mL/min calibrated mass flow controllers.

## 3. Performance of the Instrument

**Figure 3:**
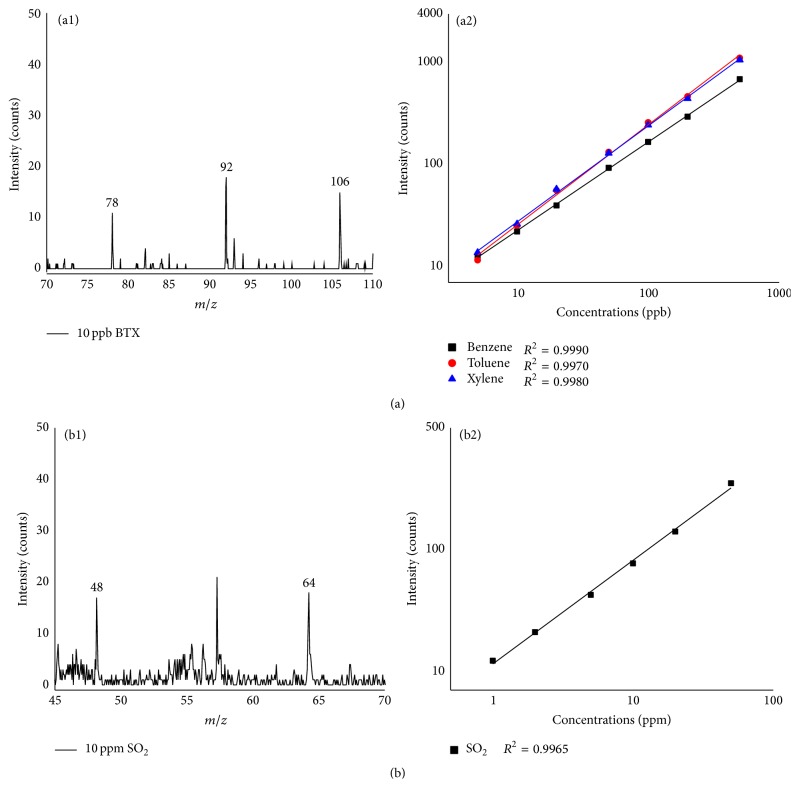
Spectra and the linear response curve for (a) benzene, toluene, and xylene and (b) SO_2_.

The detection of 10 ppb benzene, toluene, and xylene (BTX) was achieved in SPI mode with the membrane inlet. The spectra shown in Figure 3(a) were collected during 50 s at a repetition rate of 20 kHz. The linear response curve for of benzene, toluene, and xylene range is in the concentration range from 5 ppb to 400 ppm, and the linear dynamic ranges are greater than four orders of magnitude with a good linear correlation coefficient (*R* ^2^ > 0.9900). Based on the criteria of signal-to-noise ratio (*S*/*N* = 3), the limit of detection for BTX is calculated to be 1 ppbv. This sensitivity is even much better than the reported results in the literature with more powerful VUV light source [22].

SO_2_ can rarely be ionized in SPI mode for its high ionization energy (12.5 eV); therefore, PEI mode was selected for its analysis. The concentrations of SO_2_ used in the experiment were 1 ppm, 2 ppm, 5 ppm, 10 ppm, 20 ppm, and 50 ppm, respectively. The samples were introduced to the TOFMS by the capillary inlet. The spectra were collected during 75 s at a repetition rate of 40 kHz.  Figure 3(b) exhibits the mass spectrum and the linear response curve of SO_2_ for 6 different concentrations. The linear correlation coefficient was calculated as 0.9965 (*R* ^2^ = 0.9965), and the limit of quantitation (LOQ) was determined as 1 ppm based on the criteria of *S*/*N* = 10 : 1.

The mass resolving power of the portable TOFMS, defined as *M*/Δ*M*, where *M* is the peak center and Δ*M* is the full-width half-maximum of the fitted Gaussian peak, was greater than 1100 at 64* m/z* (SO_2_^+^). The obtained resolution is higher than the reported 500 resolutions with similar size of 53 × 33 × 21 cm [14]. In the laboratory, a mass resolving power of 1100 is routinely achieved for the range considered (10–300 Th). The stability of the portable TOFMS was investigated by comparing the tertian signal intensity of a 100 ppb BTX, and the relative standard deviation (RSD) was less than 10%.

## 4. Applications 

### 4.1. Rapid Analysis of VOCs in Wine

**Figure 4:**
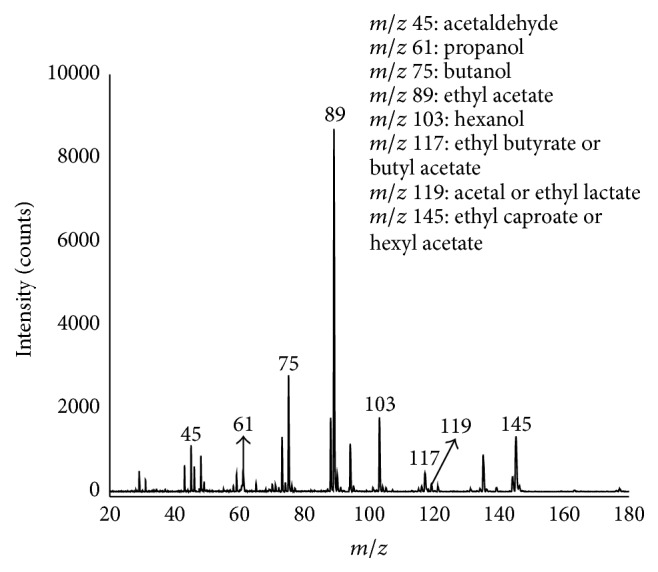
Spectrum of VOCs from wine with SPI TOFMS.

Chinese wine contains lots of trace volatile organic compounds. The portable TOFMS analyzed the headspace compounds of the wine at room temperature. The capillary inlet was plugged directly into the headspace bottle. The spectrum of VOCs from wine with SPI ionization is shown in Figure 4, and the components were interpreted as acetaldehyde (*m/z* 44, C_2_H_4_O), methanol (*m/z* 32.04, CH_4_O), n-propanol (*m/z* 62.10, C_3_H_8_O), n-butanol (*m/z* 74.12, C_4_H_10_O), ethyl acetate (*m/z* 88.11, C_4_H_8_O_2_), pentanol (*m/z* 88.15, C_5_H_12_O), furfural (*m/z* 96.08, C_5_H_4_O_2_), hexanol (*m/z* 102.15, C_6_H_14_O), ethyl butyrate (*m/z* 116.16, C_6_H_12_O_2_), aldehyde acetal (*m/z* 118.17, C_6_H_14_O_2_), ethyl lactate (*m/z* 118.13, C_5_H_10_O_3_), and ethyl hexanoate (*m/z* 144.21, C_8_H_16_O_2_). The main characteristic compounds inside of the wine were all detected by the portable TOFMS. The peaks for wine obtained with VUV lamp are characteristic with [M − H]^+^, which is quite different with [M + H]^+^ peaks by PTRMS [23].

### 4.2. Discharging Products of Sulfur Hexafluoride (SF_6_)

**Figure 5:**
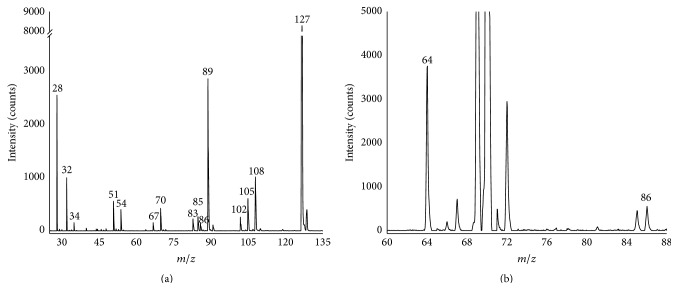
(a) Spectrum of the discharging products of SF_6_ with online PEI mass spectrometry and (b) spectrum of in situ detection of the real decomposition products of SF_6_.

Sulfur hexafluoride (SF_6_) is widely used in gas-insulated switchgear (GIS) and transformers due to its excellent insulating and arc-suppression properties. However, in the presence of partial discharge, SF_6_ decomposes into various byproducts, which leads to a significant decrease in the electric property of SF_6_. These byproducts are often used to detect and identify partial discharge. For instance, SO_2_, SOF_2_, SO_2_F_2_, SF_4_, SOF_4_, and S_2_F_10_O could be present in SF_6_ insulating devices with discharging fault. The main characteristics of these discharging products were given in Table 2. All of these compounds have higher ionization energies than 10.6 eV. Therefore, PEI mode was selected for the analysis. The capillary inlet was used and the analysis time was 13 s. The spectrum of the discharging products of SF_6_ is shown in Figure 5(a). Peaks of discharging products, SO_2_F^+^ (*m/z*, 83), SiF_3_^+^ (*m/z*, 85), SOF_2_^+^ (*m/z*, 86), SO_2_F_2_^+^ or S_2_F_2_^+^ (*m/z*, 102), and SOF_3_^+^ (*m/z*, 105), can be observed clearly in the figure. And the fragments and reaction products of SF_6_ such as SF^+^ (*m/z*, 51), OF_2_^+^ (*m/z*, 54), SF_2_^+^ (*m/z*, 70), SF_5_^+^ (*m/z*, 127), SF_4_^+^ (*m/z*, 108), and SF_3_^+^ (*m/z*, 89) can be observed as well. Peaks of O_2_^+^ (*m/z*, 32) and N_2_^+^ (*m/z*, 28) can be observed due to the air mixed with SF_6_. The portable TOFMS was used for in situ detection of the real decomposition products of SF_6_ which were sampled from an insulation fault of a GIS. The spectrum is shown in Figure 5(b); the peaks of* m/z* 64 and* m/z* 86 can be ascribed to SO_2_^+^ and SOF_2_^+^, which originate from gaseous byproducts of SO_2_ and SOF_2_, separately. The concentration of SO_2_ is calculated as 400 ppm from the linear curve, and this result indicated that serious discharging has occurred inside the GIS.

## 5. Conclusion

A VUV lamp based portable TOFMS has been developed for field analysis of gas analytes. The portable TOFMS is equipped with two sampling inlets and dual ionization operating mode for different requirements. The TOFMS is 40 × 31 × 29 cm in size and 27 kg in weight with a favorable power consumption of 70 W. Instrument performance was assessed using BTX and SO_2_, a mass resolution of 1100 at* m/z* 64 has been obtained, and, under optimized conditions, the detection limits for BTX were 0.005–400 ppm by volume with linear dynamic ranges greater than four orders of magnitude. The portable TOFMS was used to detect SO_2_, the typical decomposition product of SF_6_, for diagnosis of potential fault in SF_6_-insulated switchgear. The results reported here indicate that compact, high-performance TOFMS is ready for field development.

## Figures and Tables

**Table 1:** Typical parameters of the portable time-of-flight mass spectrometer.

Parameters	SPI mode	PEI mode
Pressure inside of ion source	1.25 Pa	1.25 Pa
Electrode 1	20 V	22.5 V
Electrode 2	17 V	21 V
Skimmer	5 V	−45.5 V
Electrode 3	−20 V	9 V
Electrode 4	−120 V	−120 V
Electrode 5	40 V	0 V
VUV lamp voltage	−1400 V	−1400 V
Repeller voltage	400 V	400 V
Accelerator	1800 V	1800 V
Reflector 1	1000 V	1000 V
Reflector 2	420 V	400 V
MCP detector	2600 V	2600 V
Pressure inside of mass analyzer	5 × 10^−5^ Pa	5 × 10^−5^ Pa

**Table 2:** Physical properties and mass-to-charge ratios (*m*/*z*) of decomposition products from SF_6_.

Compounds	IP (ev)	*M*_*w*_	Product ions
SF_6_	15.30	146	127, 108, 89
SOF_4_	12.8	124	105
SiF_4_	15.70	104	85
SO_2_F_2_	13.3	102	102
SOF_2_	12.58	86	86
SO_2_	12.35	64	64
